# Concurrent or layered treatment with radium-223 and enzalutamide or abiraterone/prednisone: real-world clinical outcomes in patients with metastatic castration-resistant prostate cancer

**DOI:** 10.1038/s41391-020-0236-0

**Published:** 2020-05-13

**Authors:** Neal Shore, Celestia S. Higano, Daniel J. George, Cora N. Sternberg, Fred Saad, Bertrand Tombal, Kurt Miller, Jan Kalinovsky, XiaoLong Jiao, Krishna Tangirala, Oliver Sartor

**Affiliations:** 1 Carolina Urologic Research Center, Myrtle Beach, SC USA; 2 Department of Medicine, University of Washington and Fred Hutchinson Cancer Research Center, Seattle, WA USA; 3 Departments of Medicine and Surgery, Duke Cancer Institute, Duke University, Durham, NC USA; 4 Weill Cornell Department of Medicine, New York-Presbyterian Hospital, New York, NY USA; 5 University of Montreal Hospital Center, Montreal, QC Canada; 6 Division of Urology, IREC, Cliniques Universitaires Saint Luc, UCLouvain, Brussels, Belgium; 7 Charité Universitätsmedizin Berlin, Urologische Klinik und Hochschulambulanz, Berlin, Germany; 8 Bayer Consumer Care AG, Basel, Switzerland; 9 Bayer HealthCare Pharmaceuticals, Whippany, NJ USA; 10 Tulane Cancer Center, Tulane University School of Medicine, New Orleans, LA USA

**Keywords:** Prostate cancer, Cancer therapy, Outcomes research

## Abstract

**Background:**

In this study, we evaluated real-world data on radium-223 plus abiraterone/prednisone or enzalutamide. Previously, the ERA 223 trial (NCT02043678) demonstrated increased fracture risk with concurrent treatment with radium-223 and abiraterone plus prednisone/prednisolone in patients with metastatic castration-resistant prostate cancer (mCRPC).

**Methods:**

We used the Flatiron Health database to perform a retrospective study of patients with mCRPC treated with radium-223. Treatment with radium-223 plus abiraterone/prednisone or enzalutamide was defined as concurrent if both drugs started within 30 days of one another, or layered when the second drug started ≥30 days after the first. The index date was defined as the day of the first radium-223 dose. Outcome measures included symptomatic skeletal events (SSEs), overall survival (OS), and patterns of treatments received.

**Results:**

Of the 625 patients treated with radium-223, 22% received it together with abiraterone/prednisone and 27% with enzalutamide. When these agents were combined, they were often initiated in a layered fashion (73% layered, 23% concurrent). Prior or concomitant bone health agents (BHAs) were received by 67% and 55% of patients, respectively. Median follow-up was 9 months. Overall, incidence rates for SSEs and pathologic fractures were 0.35 and 0.11 patients per person-year, respectively. Median OS from mCRPC diagnosis was 28.1 months.

**Conclusions:**

In this real-world setting, combination treatments with radium-223 and abiraterone/prednisone or enzalutamide were common. These agents were more commonly given in a layered than a concurrent fashion. Incidence rates for SSEs were reduced when BHAs were used; however, BHAs were underutilized.

## Introduction

The approval of several agents that prolong survival in patients with metastatic castration-resistant prostate cancer (mCRPC) has provided physicians with the opportunity to select treatment based on patient preference, disease characteristics, accessibility, cost, and clinician experience [1–11]. Because these agents have different mechanisms of action, strategies for combining or sequencing them may provide improved outcomes for select patients; however, prospective data supporting the efficacy and safety of these strategies are currently limited.

Radium-223 is a targeted alpha therapy that prolonged overall survival (OS) and time to first symptomatic skeletal event (SSE; defined as occurrence of new symptomatic pathologic fractures, spinal cord compression, external beam radiation therapy [EBRT] to relieve bone pain, or orthopedic surgical intervention) in patients with mCRPC and bone metastases in the phase 3 ALSYMPCA trial [8, 12]. Early- or expanded-access programs reported that radium-223 could be safely used in combination with abiraterone/prednisone or enzalutamide in patients with mCRPC [13–15]. However, findings from the phase 3 ERA 223 trial indicated that concurrent treatment with abiraterone/prednisone and radium-223 did not prolong SSE-free survival but rather led to an increase in the incidence of fractures in uninvolved bone compared with abiraterone/prednisone [16].

As the treatment landscape for mCRPC continues to change with the emergence of new phase 3 data, there remains a lack of information regarding treatment patterns and outcomes in the real-world setting. Consequently, treatment decisions are often based on personal clinical judgment and expert opinion. A retrospective real-world study of patients with mCRPC in the US using the Flatiron Health database showed a substantial variation in the choice of first-, second-, and third-line treatments for mCRPC, with abiraterone/prednisone, enzalutamide, and docetaxel being the most common treatment options in each respective setting. Combination regimens containing radium-223 were frequently used in the second- and third-line settings [17].

To expand on the findings from clinical trials and to provide further insights into the real-world use of combination therapies with radium-223 and abiraterone/prednisone or enzalutamide, retrospective analyses were performed using the Flatiron Health database. Two treatment approaches were evaluated: a concurrent approach (treatments starting within 30 days of each other) [18] and a layered approach (the second treatment starting ≥30 days after the first) [18]. Because bone metastases occur in ~90% of patients with mCRPC [11] and are associated with an increased rate of SSEs, which reduce patient quality of life and life expectancy [8, 12, 19, 20], the rate of SSEs was chosen as the primary objective.

## Materials and methods

A retrospective study of patients whose health records were included in the Flatiron Health database was conducted. This longitudinal database, which is demographically and geographically diverse, is derived from structured and unstructured de-identified electronic health record data, curated via technology-enabled abstraction. As of June 2019, the Flatiron database included de-identified data from over 280 cancer clinics across the US (~800 sites of care), representing more than 2.2 million patients with cancer. Data from the subset of patients with mCRPC who were treated with radium-223 were used to examine real-world outcomes with this agent, including treatment patterns, skeletal events, and survival. A general description of the Flatiron Health database and the methodology for analyzing the mCRPC subset of this database, together with a detailed description of the methods for data gathering (including patient selection) and handling (including statistical analyses), have been published elsewhere [21].

### Study design and patients

Records of patients with a confirmed diagnosis of mCRPC who were treated with radium-223 between January 1, 2013, and June 30, 2017, were included in the overall cohort. Records were excluded if patients participated in any clinical trial or had missing start dates for radium-223, enzalutamide, and abiraterone/prednisone.

For the purpose of this analysis, a concurrent treatment approach was defined as both radium-223 and either enzalutamide or abiraterone/prednisone starting within 30 days of each other, whereas a layered approach was defined as one drug starting ≥30 days after the other, giving four sub-cohorts in total [18]. The index date was defined as the date of the first dose of radium-223 and follow-up continued until death or the last data entry.

### Study objectives

The primary objective was to assess the rate of SSEs (recorded as symptomatic spinal cord compression, pathologic fracture as per investigator assessment, EBRT to the site of bone metastasis, or bone surgery). Secondary objectives were to assess OS from diagnosis of mCRPC and from initiation of radium-223. Exploratory objectives were to describe treatment patterns and duration of treatment with radium-223, enzalutamide, or abiraterone/prednisone.

### Statistical analyses

Descriptive statistical analyses were used for baseline patient demographics and disease characteristics, prior therapies, bone health agent (BHA) use, and clinical outcomes (including SSE rates and OS). The Kaplan–Meier method was used for time-to-event analyses (OS). No formal comparisons between sub-cohorts were conducted. To account for variations in follow-up time, incidence rates for SSE and pathologic fracture (number of patients per person-year) were calculated. Statistical analysis was performed using SAS Enterprise Guide Version 7.12.

## Results

### Baseline demographics and disease characteristics

The dataset included 625 patients with mCRPC who had received at least one dose of radium-223 outside of a clinical trial setting. Forty-six patients had missing administration dates for radium-223, abiraterone/prednisone, or enzalutamide and were excluded only from the sub-cohort analysis. A total of 303/625 patients (48%) received radium-223 in combination with either abiraterone/prednisone or enzalutamide. Layered or concurrent treatment with radium-223 and abiraterone/prednisone was administered to 136/625 patients (22%), and radium-223 and enzalutamide to 167/625 patients (27%). Of those who received combination treatment, most (220/303; 73%) received it as a layered regimen.

The median age of patients at the start of treatment was 73 years (Table 1). The median time from CRPC diagnosis to the start of radium-223 therapy was 11 months in the overall cohort, but varied in the sub-cohorts (≤5 months for concurrent therapy in both abiraterone and enzalutamide sub-cohorts and 10 and 14 months for layered therapy in the abiraterone and enzalutamide sub-cohorts, respectively) (Table 2). Most patients had received one or more prior life-prolonging therapies (abiraterone/prednisone, enzalutamide, docetaxel, cabazitaxel, or sipuleucel-T) and 67% of patients overall had received prior BHAs, with some variation across sub-cohorts (Table 1).

**Table 1:** Patient baseline characteristics.

	Radium-223 + abiraterone/prednisone (*n* = 136)		Radium-223 + enzalutamide (*n* = 167)		All radium-223-treated patients (*N* = 625)
	Concurrent^a^ (*n* = 39)	Layered^a^ (*n* = 97)	Concurrent^a^ (*n* = 44)	Layered^a^ (*n* = 123)	
Median age, years (min, max)	69 (55, 85)	75 (49, 85)	75 (47, 85)	71 (44, 85)	73 (44, 85)
Median ALP, U/L (min, max)	131^b^ (35, 689)	103^c^ (35, 1964)	134^d^ (51, 1964)	88^e^ (34, 1063)	108^f^ (31, 1964)
Median PSA, ng/mL (min, max)	29^g^ (1, 2040)	26^c^ (0, 1207)	32^h^ (0, 895)	26^i^ (0, 1709)	38^j^ (0, 6079)^k^
Median time from CRPC diagnosis to radium-223 initiation, months (min, max)	3 (−1, 49)^l^	10 (−4, 54)^m^	5 (0, 37)	14 (−3, 79)^n^	11 (−8, 129)^o^
Prior therapies, *n* (%)^p^					
Abiraterone/prednisone	NA	NA	15 (34)	60 (49)	344 (55)
Enzalutamide	12 (31)	31 (32)	NA	NA	335 (54)
Docetaxel	12 (31)	24 (25)	10 (23)	23 (19)	164 (26)
Cabazitaxel	1 (3)	3 (3)	1 (2)	3 (2)	36 (6)
Sipuleucel-T	6 (15)	7 (7)	2 (5)	15 (12)	62 (10)
Prior BHAs, *n* (%)					
Any BHA	26 (67)	68 (70)	30 (68)	78 (63)	419 (67)
Denosumab	17 (44)	49 (51)	16 (36)	61 (50)	288 (46)
Zoledronic acid	8 (21)	19 (20)	14 (32)	17 (14)	128 (20)
Prior SSEs, *n* (%)					
Any SSE	20 (51)	47 (48)	19 (43)	71 (58)	314 (50)
Pathologic fractures	4 (10)	13 (13)	8 (18)	22 (18)	110 (18)
Spinal cord compression	1 (3)	5 (5)	0	9 (7)	26 (4)
Surgery to bone	3 (8)	7 (7)	3 (7)	9 (7)	39 (6)
EBRT	20 (51)	42 (43)	15 (34)	62 (50)	271 (43)

*ALP* alkaline phosphatase, *BHA* bone health agent, *CRPC* castration-resistant prostate cancer, *EBRT* external beam radiation therapy, *NA* not applicable, *PSA* prostate-specific antigen, *SSE* symptomatic skeletal event.

^a^Concurrent = both treatments starting within 30 days of each other; layered = one treatment starting ≥30 days after the other.

^b^*n* = 31.

^c^n = 77.

^d^n = 35.

^e^n = 97.

^f^n = 495.

^g^n = 32.

^h^n = 37.

^i^n = 95.

^j^n = 483.

^k^Five patients with PSA > 10,000 were excluded.

^l^One patient started radium-223 earlier than the diagnosis of CRPC.

^m^Two patients started radium-223 earlier than the diagnosis of CRPC.

^n^Four patients started radium-223 earlier than the diagnosis of CRPC.

^o^Twenty-two patients started radium-223 earlier than the diagnosis of CRPC, and continued radium-223 treatment afterwards.

^p^Patients could have received more than one prior therapy.

**Table 2:** Summary of SSEs and BHA use.

	Radium-223 + abiraterone/prednisone (*n* = 136)		Radium-223 + enzalutamide (*n* = 167)		All radium-223-treated patients (*N* = 625)^b^
	Concurrent^a^ (*n* = 39)	Layered^a^ (*n* = 97)	Concurrent^a^ (*n* = 44)	Layered^a^ (*n* = 123)	
Median follow-up time, months (range)	13 (0–40)	10 (0–42)	12 (1–40)	10 (0–32)	9 (0–46)
SSEs, *n* (%)					
Any SSE	14 (36)	22 (23)	9 (20)	35 (28)	168 (27)
Pathologic fracture	7 (18)	8 (8)	4 (9)	15 (12)	61 (10)
Spinal cord compression	4 (10)	4 (4)	0	4 (3)	30 (5)
Surgery to bone	1 (3)	3 (3)	0	2 (2)	14 (2)
EBRT	10 (26)	18 (19)	7 (16)	24 (20)	132 (21)
Concomitant BHAs,^c^ *n* (%)					
Any BHA	24 (62)	59 (61)	29 (66)	71 (58)	343 (55)
Denosumab	17 (44)	44 (45)	21 (48)	60 (49)	250 (40)
Zoledronic acid	7 (18)	16 (16)	9 (20)	12 (10)	95 (15)

*BHA* bone health agent, *EBRT* external beam radiation therapy, *SSE* symptomatic skeletal event.

^a^Concurrent = both treatments starting within 30 days of each other; layered = one treatment starting ≥30 days after the other.

^b^Two patients had no follow-up data and were excluded from the analysis.

^c^Concomitant BHA therapy was defined as the presence of a BHA at any time during radium-223 therapy. A patient could have received more than one BHA during radium-223 therapy, and the use of BHAs could have occurred before or after an SSE (no causality is implied).

At baseline, 314/625 patients (50%) in the overall cohort had documented prior SSEs, with similar proportions across all four sub-cohorts. In the overall cohort, 110/625 patients (18%) experienced prior pathologic fractures, compared with 10% and 13% in the concurrent and layered abiraterone/prednisone sub-cohorts, respectively, and 18% in the enzalutamide sub-cohorts (Table 1).

### Outcomes

The median follow-up time was 9 months for the overall cohort and 10–13 months across all four sub-cohorts (Table 2).

#### Symptomatic skeletal events (SSEs)

A total of 168/625 patients (27%) in the overall cohort had experienced an on-treatment SSE, ranging from 20 to 36% across each of the four sub-cohorts (Table 2). The most frequent SSE was EBRT use for pain palliation in 21% of the overall cohort and 16–26% of the sub-cohorts (Table 2). Pathologic fractures were reported in 10% of patients in the overall cohort, varying from 8% in the layered radium-223 and abiraterone/prednisone sub-cohort to 18% in the concurrent radium-223 and abiraterone/prednisone sub-cohort (Table 2).

**Fig. 1:**
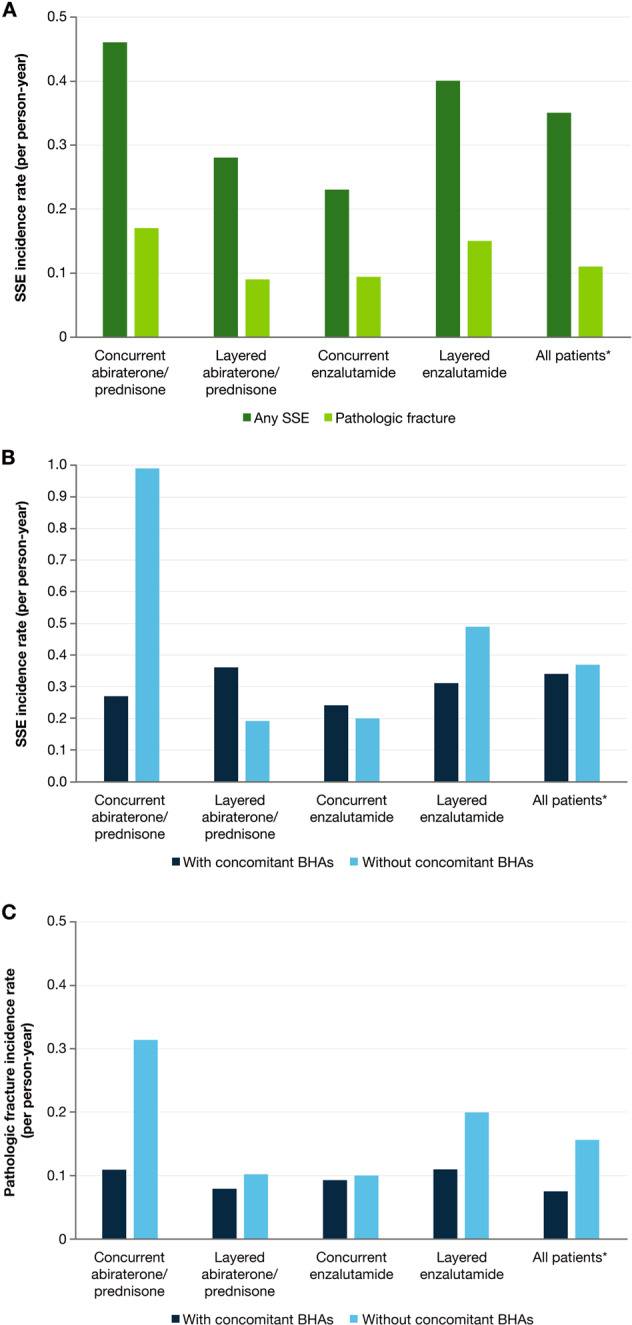
SSE and pathologic fracture incidence rates. **a** Overall SSE and pathologic fracture incidence rates. **b** SSE incidence rates, stratified by concomitant BHA usage. **c** Pathologic fracture incidence rates, stratified by concomitant BHA usage. Concurrent = both treatments starting within 30 days of each other; layered = one treatment starting ≥30 days after the other. BHA bone health agent, SSE symptomatic skeletal event. *Two patients had no follow-up data and were excluded from the analysis.

Incidence rates for SSEs and pathologic fractures were 0.35 and 0.11 patients per person-year, respectively, in the overall cohort. Variations in SSE rates were observed across sub-cohorts, ranging from 0.23 in the concurrent radium-223 and enzalutamide sub-cohort to 0.46 in the concurrent radium-223 and abiraterone/prednisone sub-cohort (Fig. 1a). Pathologic fracture incidence rates also varied, ranging from 0.09 in the layered radium-223 and abiraterone/prednisone sub-cohort to 0.17 in the concurrent radium-223 and abiraterone/prednisone sub-cohort (Fig. 1a).

Incidence rates for SSEs across the four sub-cohorts were also analyzed according to whether patients had experienced prior SSEs. Incidence rates ranged from 0.14 to 0.47 patients per person-year for the subgroups with no prior SSEs and 0.29 to 0.47 patients per person-year for the subgroups who had experienced prior SSEs.

Overall, 40% and 15% of patients had received concomitant denosumab and/or zoledronic acid, respectively (Table 2). Incidence rates for SSEs in the overall cohort were 0.34 and 0.37 in patients with and without concomitant BHA use, respectively. Incidence rates for SSEs ranged from 0.24 to 0.36 across sub-cohorts in patients with concomitant BHA use and 0.19 to 0.99 for patients without concomitant BHA use (Fig. 1b). Incidence rates for pathologic fractures in the overall cohort were 0.07 and 0.15 in patients with and without concomitant BHA use, respectively, and ranged from 0.08 to 0.11 across sub-cohorts in patients with concomitant BHA use and from 0.1 to 0.31 across sub-cohorts in patients without concomitant BHA use (Fig. 1c).

#### Overall survival

**Fig. 2:**
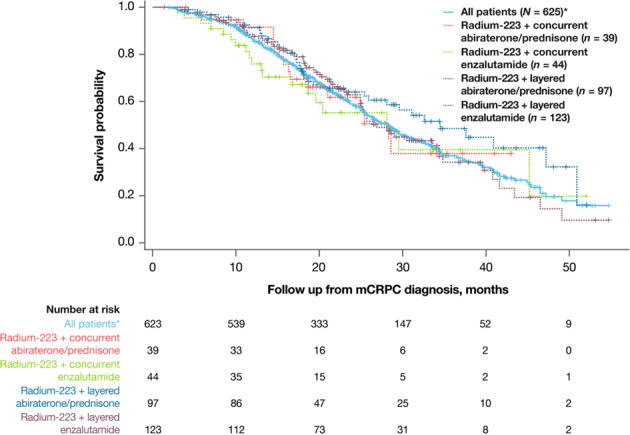
Overall survival. mCRPC metastatic castration-resistant protate cancer. Concurrent = both treatments starting within 30 days of each other; layered = one treatment starting ≥30 days after the other. *Two patients had no follow-up data and were excluded from the analysis.

Median OS from diagnosis of mCRPC was 28.1 months in the overall cohort and ranged across sub-cohorts from 28.1 to 34.5 months (Fig. 2 and Table 3). Median OS from radium-223 initiation was 15.2 months in the overall cohort and ranged from 15.2 to 22.1 months across sub-cohorts (Table 3).

**Table 3:** Summary of overall survival.

	Radium-223 + abiraterone/prednisone (*n* = 136)		Radium-223 + enzalutamide (*n* = 167)		All radium-223-treated patients (*N* = 625)^b^
	Concurrent^a^ (*n* = 39)	Layered^a^ (*n* = 97)	Concurrent^a^ (*n* = 44)	Layered^a^ (*n* = 123)	
Median follow-up time, months (range)	13 (0–40)	10 (0–42)	12 (1–40)	10 (0–32)	9 (0–46)
Median OS, months (95% CI)					
From mCRPC diagnosis	28.3 (18.4, NR)	34.5 (25.9, 50.9)	28.1 (16.7, NR)	26.9 (25.0, 34.4)	28.1 (25.7, 30.4)
From radium-223 initiation	22.1 (14.7, NR)	19.3 (11.3, 27.5)	19.1 (12.3, NR)	15.2 (11.6, 16.3)	15.2 (13.2, 16.3)

*CI* confidence interval, *mCRPC* metastatic castration-resistant prostate cancer, *NR* not reached, *OS* overall survival.

^a^Concurrent = both treatments starting within 30 days of each other; layered = one treatment starting ≥30 days after the other.

^b^Two patients had no follow-up data and were excluded from the analysis.

## Discussion

The increase in the number of available treatment options for mCRPC has considerably improved outcomes for patients. However, optimal sequences or combinations of these agents have yet to be fully determined, and real-world experience can provide valuable insights into treatment patterns and outcomes. This retrospective study evaluated real-world clinical practice data from the Flatiron Health database of 625 patients with mCRPC treated with radium-223 between 2013 and 2017, primarily in a community practice setting in the US. The Flatiron Health database comprises a large sample of patients with wide geographical coverage within the US, providing a representative sample of patients with mCRPC in clinical practice. Consequently, this database provides a robust source of information about treatment patterns and outcomes in the community setting in the US.

The results of the phase 3 ERA 223 trial showed an increased incidence of bone fractures and no improvement in SSE-free survival or OS in patients who received radium-223 in combination with abiraterone/prednisone compared with abiraterone/prednisone [16]. In our real-world analysis, which represents treatment patterns prior to the availability of ERA 223 findings, approximately half of the overall cohort had received a combination regimen of radium-223 and either abiraterone/prednisone or enzalutamide, concurrently or in a layered fashion, despite a lack of prospective data from randomized and controlled large phase 3 trials supporting these combinations. This finding is similar to that reported in another real-world study of US patients with mCRPC [22].

Previously, the safety and tolerability of combination regimens with radium-223 were evaluated in the open-label, phase 2 eRADicAte and enzaRADiCate trials [23, 24]. In these studies, concurrent administration of radium-223 with either abiraterone/prednisone or enzalutamide was investigated in patients with symptomatic mCRPC and bone metastases. In both studies, these combination regimens appeared to provide clinical benefit without evidence of increased toxicity [23, 24]. However, the phase 3 ERA 223 trial findings did not support this combination, as a greater proportion of patients had at least one fracture in the radium-223 plus abiraterone/prednisone arm than in the abiraterone/prednisone arm (19% vs. 6%, respectively). Among those patients with at least one fracture, the proportion of pathologic fractures (presence of bone metastases at the site of fracture) was ~26% in both arms. However, the proportion of osteoporotic fractures was 49% in patients in the radium-223 plus abiraterone/prednisone arm and 17% in the abiraterone/prednisone arm. These variations in the incidence of different types of fractures highlight the challenges with interpreting fracture incidence across different studies, and the importance of understanding whether a fracture is pathologic or osteoporotic [16, 25]. In our retrospective real-world analysis, the overall incidence of pathologic fractures was 10%, although there were variations across sub-cohorts, ranging from 18% to 8% in the concurrent and layered radium-223 plus abiraterone/prednisone arms, respectively. In comparison, the overall incidence of fractures was 10% in the PROSPER, PREVAIL, AFFIRM, and 97850-CL-0232 trials of enzalutamide [26], and 15% in the COU-AA-301 trial of abiraterone/prednisone [27]; fracture rates of 11% were reported in the abiraterone/prednisone plus placebo arm of the ERA 223 trial [16]. It is possible that less-frequent imaging in the real-world setting may have led to reported fractures rates being lower than those reported in phase 3 trials, or that some fractures were deemed not to be clinically relevant. The result may also be affected by varied follow-up time at the patient level. Non-pathologic fractures, as defined in the ERA 223 trial, were not captured in our real-world data. Most importantly, in this analysis, 67% and 55% of patients had received prior and concomitant BHAs, respectively, whereas in the ERA 223 trial, 41% of patients were on BHAs at study entry and subsequent initiation of BHAs was not permitted [16].

Our findings provide insights into effectiveness and safety outcomes of radium-223 in combination regimens in a real-world setting, which complement and expand on the findings from controlled clinical trials like ERA 223. In our real-world data, patients had varying degrees of disease severity and had received a range of prior therapies, including chemotherapy and other life-prolonging therapies, representing a more generalized patient population. In ERA 223, eligibility criteria defined patients earlier in the course of mCRPC; namely, those who were asymptomatic or minimally symptomatic and who had received no prior chemotherapy, abiraterone/prednisone, or enzalutamide [16]. In contrast to the ERA 223 population, half of the patients in our real-world cohort had experienced SSEs prior to commencing radium-223 therapy, suggesting a delay in the initiation of radium-223 treatment, and almost one-fifth of the patients had prior pathologic fractures. Furthermore, patients in the real-world setting had received radium-223 in either a concurrent or layered fashion, whereas patients in the ERA 223 trial received a concurrent regimen. Of the 303 patients who received a combination regimen in this real-world analysis, a higher proportion received it in a layered (73%) rather than a concurrent fashion (27%).

The ongoing observational REASSURE trial, evaluating the long-term safety of radium-223 in patients with mCRPC and bone metastases, should provide further insights into the safety and tolerability of these combination regimens in a real-world setting. Results from the first interim analysis indicate that the safety profile of radium-223 in patients with or without prior chemotherapy, and with or without prior or concomitant use of abiraterone/prednisone or enzalutamide, did not reveal any new safety signals, although some drug-related adverse events were more commonly reported in patients who had received prior therapies and had a higher disease burden [28, 29].

The phase 3, multicenter, randomized EORTC 1333/PEACE III trial (NCT02194842) is investigating the efficacy and safety of concurrent treatment with radium-223 and enzalutamide compared with enzalutamide alone as first-line therapy in patients with asymptomatic or mildly symptomatic, chemotherapy-naïve mCRPC, and bone metastases. An interim safety analysis showed that although the risk of fractures was higher when enzalutamide was given in combination with radium-223 than when enzalutamide was given alone, this risk was almost eliminated when continuous BHA use was mandated [30]. In combination with the findings from ERA 223, these data highlight the importance of using BHAs when treating patients with the combination of radium-223 and either abiraterone/prednisone or enzalutamide. Similarly, in the ERA 223 trial, the proportion of patients who had fractures in either treatment arm (abiraterone/prednisone with or without radium-223) was lower for patients taking BHAs than those who were not [16]. Interim findings from the REASSURE study have shown that BHAs were underutilized in patients with mCRPC [31]. In this real-world study, approximately half of all patients received BHAs, in line with the proportions reported in phase 3 trials and the REASSURE observational study [8, 16, 31, 32]. Patients treated with BHAs had a lower incidence of pathologic fractures compared with those who did not receive concomitant BHAs, consistent with the findings of the phase 3 trials of zoledronic acid and denosumab [33, 34]. These findings from both clinical trials and the real-world setting highlight the fact that BHAs are underutilized in this patient population and that guideline recommendations for the use of BHAs in mCRPC are not entirely followed. It is clear that following the guidelines could reduce the incidence of fractures in patients with mCRPC, irrespective of treatment received.

Limitations of our study include that, while the Flatiron Health network has broad coverage, it does not include urologists or urology centers, and it is unknown if it fully reflects prostate cancer care and outcomes. In addition, the study was descriptive in nature, so no formal statistical comparisons were made between groups, in part because of small sample sizes; thus, there were no adjustments for potentially confounding factors. Regarding treatment, the dates on which treatment was discontinued may not always have been properly recorded. For oral therapies, data were derived both from prescriptions and pharmacy orders in the electronic health record, but whether these prescriptions were filled or refilled cannot be verified, so analysts may have considered a business rule that extended the use of an oral therapy to the start date of the next therapy. The median follow-up time of 9 months for the overall cohort and 10–13 months across all four sub-cohorts is dependent on the median OS of this patient population, and therefore is unlikely to exceed 1 year unless long-term survival increases in this patient population over the observation period. However, the observation period for the study commenced in 2013, meaning that data were collected over several years. Furthermore, there is a potential survival bias for OS, because the treatment groups in this study are defined by the treatments received after mCRPC diagnosis, but time zero is diagnosis of mCRPC. Thus, all patients had to survive long enough to receive treatment.

In summary, in this retrospective study of real-world data from patients with mCRPC who received radium-223, combination therapy with radium-223 and either abiraterone/prednisone or enzalutamide was common. Layered therapy was more common than the concurrent approach used in the ERA 223 trial. Real-world data cannot be directly compared with data from phase 3 trials, but an indirect comparison shows that reported pathologic fracture rates appeared to be lower in general in the real-world setting than in clinical trials. Overall, SSE incidence rates and pathologic fracture rates were reduced with BHA use, but only 67% and 55% of patients received prior or concurrent BHAs, respectively. As experience with the use of radium-223 grows, real-world evidence can provide valuable additional information on outcomes across a range of settings to complement the findings from controlled clinical trials.
